# Genome Sequence of *Bacillus cereus* Group Phage SalinJah

**DOI:** 10.1128/genomeA.00953-16

**Published:** 2016-09-29

**Authors:** Ivan Erill, Steven M. Caruso

**Affiliations:** Department of Biological Sciences, University of Maryland, Baltimore County, Baltimore, Maryland, USA

## Abstract

The double-stranded DNA (dsDNA) *Myoviridae Bacillus cereus* group bacteriophage SalinJah was isolated from soil collected in Gyeonggi-do, South Korea. SalinJah, a cluster C phage with a broad host range, suggests the need to create a new subcluster with SalinJah and Helga as founding members.

## GENOME ANNOUNCEMENT

*Bacillus* phage SalinJah was isolated from a soil sample collected from Gyeonggi-do, South Korea (37°30′00.0″N, 127°15′00.0″E), and isolated on *Bacillus thuringiensis* Berliner 1915 DSM 350 (*Bt*-350), a nonpathogenic (1) member of the *Bacillus cereus* group, along with other endospore-forming *Bacillus* species, such as *B. cereus*, *B. anthracis*, *B. weihenstephanensis,* and others (2), and it is available from the German Collection of Microorganisms and Cell Cultures (https://www.dsmz.de/). Isolation and characterization of SalinJah were completed by undergraduate student researchers as part of the SEA-PHAGES program, as described previously (3). Additional information about SalinJah and other *Bacillus* phages isolated by undergraduate researchers can be found on the *Bacillus* phages database (BPDB) (http://bacillus.phagesdb.org/).

SalinJah is a *Myoviridae* double-stranded (dsDNA) bacteriophage with an icosahedral head 80 nm in diameter and a 187-nm contractile tail. It produces approximately 1-mm turbid plaques when grown on *Bt*-350 overnight on Trypticase soy agar. Shotgun sequencing was carried out by the Pittsburgh Bacteriophage Institute to approximately 878-fold coverage by Illumina sequencing. SalinJah has a linear 161,140-bp genome, with 2,770-bp direct terminal repeats and a G+C content of 38.7%. Genomic analysis determined that SalinJah contains 292 protein-coding genes, of which 45 have been assigned predicted functions.

A comparison of SalinJah with other *Bacillus* phages by nucleotide identity, synteny, and phylogenetic analysis identified *Bacillus* phage Helga (see BPDB) as the most similar previously sequenced phage, with an average nucleotide identity (ANI) of 0.92 (4). SalinJah and Helga share high similarity with members of cluster C, subcluster C1, such as *Bacillus* phages Hakuna (accession no. KJ489399) and Megatron (accession no. KJ489401) in the above-mentioned respects, as well as in the presence of a large noncoding region, the lack of tRNA genes, and by encoding the same group I endolysins (5). In contrast, SalinJah shows lower ANI with phages in other C subclusters, with an ANI of 0.63, 0.62, and 0.59 compared to members of subclusters C2, C3, and C4, respectively. However, the average ± standard deviation (SD) ANI among established and presumptive subcluster C1 phages is 0.92 ± 0.03, whereas the average ± SD ANI of SalinJah and Helga with C1 subcluster members is only 0.79 ± 0.01. This is below the 0.80 threshold established for subclusters (5). Together with differences observable through phylogenetic analysis and whole-genome alignment (6), this suggests the need for the creation of a new subcluster encompassing SalinJah and Helga.

As with C1 subcluster members, SalinJah demonstrated a broad host range and was able to infect several *B. cereus* group species, including *B. cereus* FDA5 (ATCC 10702) and Gibson 971 (ATCC 14579) and *B. thuringiensis* Al Hakam, PS52A1, and *Bacillus* (ATCC 33679), although not *B. anthracis* delta Sterne or more distantly related *Bacillus* species tested. Codon usage bias analysis using scnRCA (7), using *B. thuringiensis kurstaki* HD73 (accession no. NC_020238.1) as a reference strain, reveals optimization of lytic phase genes (e.g., tail and capsid proteins, RecA-type recombinases, endolysins, and the two ribonucleotide-diphosphate reductase subunits) for translational throughput on *B. cereus* strains, in agreement with previous findings for *B. cereus* group bacteriophages.

### Accession number(s).

The complete genome sequence of the *Bacillus* phage SalinJah is available in GenBank with the accession no. KX011169.

## References

[1] Schulenburg H, Müller S 2004 Natural variation in the response of *Caenorhabditis elegans* towards *Bacillus thuringiensis*. Parasitology 128:433–443. doi:10.1017/S003118200300461X. 15151149

[2] Maughan H, Van der Auwera G 2011 *Bacillus* taxonomy in the genomic era finds phenotypes to be essential though often misleading. Infect Genet Evol 11:789–797. doi:10.1016/j.meegid.2011.02.001. 21334463

[3] Jordan TC, Burnett SH, Carson S, Jordan TC, Burnett SH, Carson S, Caruso SM, Clase K, DeJong RJ, Dennehy JJ, Denver DR, Dunbar D, Elgin SCR, Findley AM, Gissendanner CR, Golebiewska UP, Guild N, Hartzog GA, Grillo WH, Hollowell GP, Hughes LE, Johnson A, King RA, Lewis LO, Li W, Rosenzweig F, Rubin MR, Saha MS, Sandoz J, Shaffer CD, Taylor B, Temple L, Vazquez E, Ware VC, Barker LP, Bradley KW, Jacobs-Sera D, Pope WH, Russell DA, Cresawn SG, Lopatto D, Bailey CP, Hatfull GF 2014 A broadly implementable research course in phage discovery and genomics for first-year undergraduate students. mBio 5:e01051-13. doi:10.1128/mBio.01051-13. 24496795 PMC3950523

[4] Hatfull GF, Jacobs-Sera D, Lawrence JG, Pope WH, Russell DA, Ko CC, Weber RJ, Patel MC, Germane KL, Edgar RH, Hoyte NN, Bowman CA, Tantoco AT, Paladin EC, Myers MS, Smith AL, Grace MS, Pham TT, O’Brien MB, Vogelsberger AM, Hryckowian AJ, Wynalek JL, Donis-Keller H, Bogel MW, Peebles CL, Cresawn SG, Hendrix RW 2010 Comparative genomic analysis of 60 mycobacteriophage genomes: genome clustering, gene acquisition, and gene size. J Mol Biol 397:119–143. doi:10.1016/j.jmb.2010.01.011. 20064525 PMC2830324

[5] Sauder AB, Quinn MR, Brouillette A, Caruso S, Cresawn S, Erill I, Lewis L, Loesser-Casey K, Pate M, Scott C, Stockwell S, Temple L 2016 Genomic characterization and comparison of seven *Myoviridae* bacteriophage infecting *Bacillus thuringiensis*. Virology 489:243–251. doi:10.1016/j.virol.2015.12.012. 26773385

[6] Cresawn SG, Bogel M, Day N, Jacobs-Sera D, Hendrix RW, Hatfull GF 2011 Phamerator: a bioinformatic tool for comparative bacteriophage genomics. BMC Bioinformatics 12:395. doi:10.1186/1471-2105-12-395. 21991981 PMC3233612

[7] O’Neill PK, Or M, Erill I 2013 scnRCA: a novel method to detect consistent patterns of translational selection in mutationally-biased genomes. PLoS One 8:e76177. doi:10.1371/journal.pone.0076177. 24116094 PMC3792112

